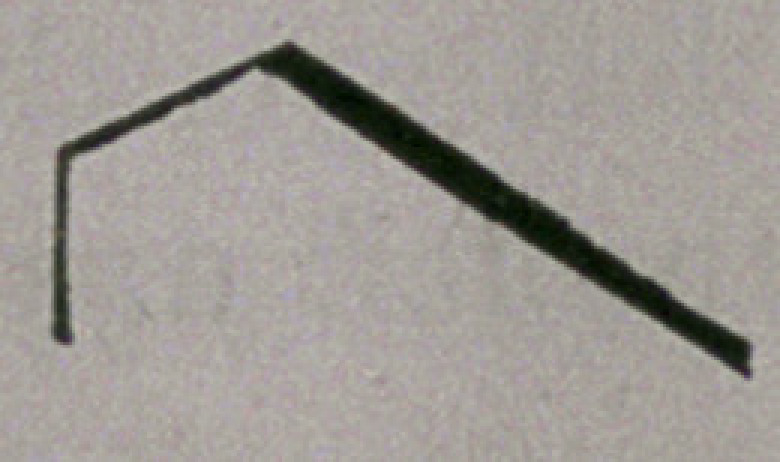# Cinchona Officinalis; Provings

**Published:** 1883-02

**Authors:** B. Fincke

**Affiliations:** Brooklyn, N. Y.


### CINCHONA OFFICINALIS.

### PROVINGS BY B. FINCKE, M. D., BROOKLYN, N. Y.

I.—B. F., 29 years old, single, of slender habit, nervous temperament, dark fine hair, gray eyes, generally healthy, took, 1850, June 27th, 2½ P. M., one drop of China regia 27 centes. Immediately smarting, acrid taste, slight vertigo.

3 P. M.—Stitch on the sole of the left foot.

3¼ P. M.—Pressing in the left big toe, in the bones.

3½ P. M.—Stinging upon the right big toe and its ball.

4½ P. M.—Twitching around the left middle foot. Stitches here and there. A pressure at the right hand. Head free again.

4¾ P. M.—Stinging in the right upper lip.

5 P. M.—Itching on the scalp.

6 P. M.—Painful twitching at the occiput.

7 P. M.—Pressing in the left middle foot, increasing and decreasing, going down to the sole of the foot, behind the toes. A stitch in the right heel. Hard pressing in the left hand, sideward, near the little finger.

7¾ P. M.—Pressing stinging in the right inner ear. Stinging pressure at the right thumb.

9½ P. M.—Twitching alternately in the right and left toes.

10 P. M.—Twitching in the left index-finger, at the tip and under the nail. Twitching in front of the left ankle. A twitch through the right side of the head, from before, backward. Itching stitches at the abdomen and knee.

June 28th.—Pleasant dream of a beloved person he has not thought of for a long time.

6½ A. M.—On waking up, twitching in the left foot; sideward on the right hand, and in the left arm inward.

6¾ A. M.—Twitch at the left upper calf. Red veins in the white of the eye.

8¼ A. M.—Twitching, pressing in the left inner ear.

8¾ A. M.—Stinging at the left lower calf. Stinging drawing twitching in the tip of the left index-finger. Pressure in the left heel.

9 A. M.—Stinging in the right groin. Pressure in the left knee. Picking in the skin of the left chest. Itching stitches on the scalp. Various stitches in the skin. Drawing pressing in the right inner hand.

$9\frac{1}{4}$ A. M.—Biting in the skin at the left shoulder. Drawing in of the tendon of the left index-finger, with heat therein in several attacks at a place where last year a furuncle had been produced by a dose of one drop of Sulphur 6th centes. Drawing in the meatus externus of the left ear. Pressing in the left buttock. Itching on the top of the head. Drawing at the left side of the tendo Achillis.

$9\frac{1}{2}$ A. M.—Drawing pressure across the forehead several times, and over the left eye. The left eye feels heavy and affected. Twitching in the tip of the left big toe at frequent intervals. Twitching in the corn of the left little toe. Biting in the tips of the middle toes.

$9\frac{3}{4}$ A. M.—Biting stinging between the first and second knuckle, going sometimes into the joint of the index-finger (the very place where the sulphur-furuncle had caused such severe burning pains). Drawing in the left middle and fourth fingers. Drawing in the right side of the head.

10 A. M.—Drawing on the sole of the left foot, in the hollow formed by the balls of the toes where an oblong callosity exists.

$10\frac{1}{4}$ A. M.—Hurried stitching on the left big toe. Pressing in the left calf. Slight straining in the left ear. Stinging on the left index-finger.

$10\frac{1}{2}$ A. M.—Sensation of lameness in the left hand. Stinging in the skin, over the navel, at the thigh, and at the left cheek, successively.

11 A. M.—Drawing pressing in the left inner hand, at the same time prickling on the skin covering it. A drawing through the anterior part of the head. Drawing in the left upper arm, at the insertion of the deltoid. Twitching straining in the right ear.

$11\frac{1}{2}$ A. M.—Jerk over the left eye, through the forehead. Jerking drawing in the bone of the left upper arm. Twitching in the tip of the left index-finger.

$11\frac{3}{4}$ A. M.—Pressing upon the second joint of the left little finger. Biting in the skin of the left side, with a chill running down as far as the calf. Twitching over the left eye, in the bone, through the forehead toward the occiput. Stinging on the left great toe. Lame-like drawing in the joints of the right index-finger. Itching on the right upper cheek.

M.—Stinging in the tip of the left middle finger, with a cold shudder all over. Pulsating pressing in the left big toe. Drawing from the right knee downward.

$\frac{1}{4}$ P. M.—Drawing in the middle joint of the left middle finger.

3½ P. M.—Stinging in the tip of the right big toe, running under the skin at the left shinbone.

3¾ P. M.—Pain like gout in the left heel when treading upon.

4 P. M.—Fine stinging at the palmar side of the left wrist. A violent stitch in the left chest. A violent stitch in the left heel. Drawing and pressing in the left big toe. A stitch in the right thumb. Heaviness of the head. Drizzling at the left lower lid. Stinging pressure in the side of the chest, in single, interrupted, slighter, and stronger pulses. Stinging between the lower jaw and neck. Violent stinging on the second toe, and then under it. Biting in the skin in the region of the shoulder, with perspiration. Picking at the floor of the left ear.

4½ P. M.—Pulsating stinging in the left big toe, and at the same time lamelike drawing pain around the inner side of the left heel. Drawing pain in the right toes, several times. Squeamishness of the stomach. Belching of wind. Yawning. Stinging in the navel. Stinging at the left ankle.

5 P. M.—Perspiration while quietly sitting and writing. Urging in the throat. Biting stinging in the left second toe. Belching of wind and yawning. Drawing in the right middle finger.

5½ P. M.—Drawing stinging in the left elbow. Conjunctiva less red than this morning. Drawing in the left inner hand. Twitching stinging in the ball of the right hand. Biting twitch at the right side of the abdomen, the right knee, and the scalp. Itching at the left side of the abdomen. A red streak, as if from a scratch, appeared about 3 P. M. at the back of the left hand, about this form, but double size, which now is no more to be seen. Continual perspiration.

5¾ P. M.—Drawing at the right dorsal side of the right hand. Stinging in the skin of the buttocks and abdomen. Head dull and heavy.

6 P. M.—Stinging at the left upper lid. Profuse perspiration. Fine pulsation and sensation in the left ear, as of something fallen before it.

6½ P. M.—Gouty pains in the right little finger. Sensation in the right eye as of a foreign body. The eyes feel much affected.

10½ P. M.—Lamelike drawing in the left part of the head. Drawing in the left zygoma. Discharge of stinking winds. Sound sleep during the night.

June 29th, 4 A. M.—Energetic seminal emission with following hunger and vivacity. Discharge of stinking winds.

8 A. M.—Lamelike drawing in the left heel and then higher up as if in the bone.

8¾ A. M.—Lamelike drawing in the whole right lower extremity as if in the bone. Twitching at the right buttock.

9 A. M.—A stitch in the left heel. Drizzling at the lower angle of the shoulder-blade.

9½ A. M.—Biting in the skin of the back.

9¾ A. M.—Biting in the skin of the right arm, and at the mons veneris.

10 A. M.—Biting itching in the prepuce. Straining in the right ear. Mucus discharge from the nose, head and eyes being free.

10¼ A. M.—Sleepiness. Heaviness in the forehead. Lamelike drawing pain of the right hand at the joints of the middle and ring fingers. Tiredness. Frequent yawning. Almost invincible sleepiness. Lamelike drawing in the left hand.

10¾ A. M.—Lamelike pain in the left knee on extending it Biting. stitches on the right second toe. Twitching in the right middle finger. Fine twitching at the knee.

11½ A. M.—Pale, sickly, sunken countenance.

M.—Lamelike in the hand in taking firm hold of anything. Drawing at the ball of the left foot, repeatedly. Head free again. Prickling itching upon the right thigh and at the prepuce. Mind cheerful and composed. Itching upon the chest. Lamelike stitches in the right hand.

¼ P. M.—Stinging on the skin of the right groin. Biting stitches at the ring of the prepuce, upon the left thigh and scalp, with cold shuddering all over the skin.

½ P. M.—Stitches at the prepuce.

2½ P. M.—Stitches at the left knee. Lamelike drawing and stinging all over the body, here and there.

3½ P. M.—Lamelike drawing at the left knee sideward.

4 P. M.—Lamelike drawing at the left thigh. Stitch in the interior of the forearm. A long stitch in the head behind the right frontal eminence. Biting at the right buttock, at the side of the chest, at the neck.

4¼ P. M.—Lamelike drawing at the left tendo Achillis.

4½ P. M.—Heaviness and tension in the fore and back head. Pressing upon the eyes. Drawing in the right knee and thigh. Lameness-pain in the right kneebend.

5 P. M.—Pressing together in the right big toe, running over the left foot. Lamelike drawing in the right index-finger. Stinging in the tip of the right index-finger. Pressing upon the left toes. Pressing in the left chest. Stinging at the ring of the prepuce, with cold shuddering along the left thigh.

6½ P. M.—Drawing and pressing upon the hand.

7¾ P. M.—Stinging and pulsating in the left chest.

9 P. M.—Stitches in the left side of the chest and at the same time in the left arm. Pressing in the left big toe. Distinct dreams in the night.

June 30th, 9 A. M.—Drawing and pressing in the left chest.

10½ A. M.—Lameness pain in the right knee, that he could not tread. Drawing in the chest several times during the day. Pressing in the ball of the end of the thumb, as after a stitch with a needle, in writing.

July 1st, A. M.—Dull drawing in the right foot and in the left shoulder in the forenoon. Stinging at the left side of the left index-finger. Pressing in the first joint of the left index-finger. Stinging pressing upon the left middle finger.

4 P. M.—Straining in the left ear. Stinging all over the fingers of the left hand. Stinging drawing in the left ankle-joint. A stitch in the right eye.

4¾ P. M.—Drawing at the back of the neck. Drawing in the forehead. Twitching straining in the left ear. Stinging in the left eye, with urgency to close it, which again causes stinging pain. Stitches in the heel in treading upon. Constipation. Stinging under the root of the nail of the left thumb. Vivid dreams during the night. Biting in the skin.

July 2d, 11 A. M.—Sensation like blisters on the middle tongue. Gums painful. Right side of the hand like lame. Keen appetite. Drawing in the tip of the right index-finger. After moving the hands or feet frequently, lamelike drawing, tearing, itching pains in the parts used.

3 P. M.—Straining in the left ear. Tongue cracked as from smoking. Troublesome biting in the skin at different parts.

5½ P. M.—Stinging in the tongue. Stinging in the right eye.

6½ P. M.—Stinging itching at the right elbow, in the armpit, and kneebend, the latter lasting awhile. Distinct dreams through the night.

July 3d, A. M.—Twitching in the hand on waking up. Lamelike drawing in the right index-finger.

6th.—Lamelike drawing, especially in the hands. Depression of spirits. Tenderness of feeling. Restlessness of mind. Sexual desire easily excited and frequent erections.

9th.—Violent drawing in the fingers of the right hand. Much sexual desire. Twitching, drawing, and stinging in the left hypochondrium until late in the evening, when the sensation resembles a lameness. Lamelike in the right hand.

10th.—Violent stitches in the anus on sitting down, the anus being tightly closed.

11th.—Painful straining and pressing in the left ear at intervals in the morning after rising, and continuing all day. Twitching pressing stinging in the left hypochondrium toward the back all day long. In spite of the changeable, rainy weather, which usually affects it, the head is free.

12th.—The last mentioned two symptoms in the left ear and in the left hypochondrium commence again this morning, mostly when at rest.

11 A. M.—The same twitching pressing stinging in the right armpit as before.

13th.—The sexual desire is entirely gone.

18th.—Inability to get rested in the morning; he wants to sleep longer.

23d, 9 A. M.—Violent lamelike drawing in the left toe-joints.

P. M.—The same in the three middle toes.

24th.—Diarrhœa in the morning, soon passing off after smelling the tincture of Pulsatilla 3. Lamelike drawing in several limbs here and there.

25th.—Very sensitive to saltish and acid food for some time past.

*Remark.*—This morning a trituration of Camphora, and in the evening one of Hyocyamus was made, and before bedtime Camphora two globules of the sixth were taken as an antidote.

The ensuing night was passed in sound sleep; toward morning an energetic seminal emission with following feeling of comfort.

July 26th.—Moisture in the left ear. Mind cheerful, inclined to work.

11 A. M.—Lamelike drawing in the left thigh on a small spot, and in the right middle finger.

July 28th.—Nausea after exertion. (Smelled Nux vom. 30 before bedtime, followed toward morning by an ugly dream of masturbation, practiced by others before his eyes, followed by a seminal emission.) After rising in the morning sleepiness; he falls asleep frequently over his writing during the forenoon.

P. M.—The twitching, pressing, stinging in the left hypochondrium (as the 11th) reappears and extends from the left side of the spine along the short ribs toward the upper abdomen. Lamelike drawing in the right hand.

Aug. 2d.—Lascivious dream with seminal emission early in the morning.

II.—The same prover.

1859. October 14th, took China regia 600 six glob. (Korsakoff centes.) at bedtime, and observed the next morning: Lamelike drawing, pressing pain at the dorsal middle part of the left forearm, passing along the tendon of the extensor digitorum communis into the middle finger, lasting ten minutes.

III.—Gottlieb Heinrich, a robust peasant boy, twelve years old, of dark complexion, told me that on preparing from the above-mentioned China 600 (and 27), the eighth thousandth centesimal potency, on the Korsakoffian plan, he felt frightful tearing pain in the middle ear, going into the temple, and thence into the forehead, causing a contractive ache in it; drawing stinging pain in the joints of the right arm, going into the fingers, and thence backward into the hand and arm.

IV.—Pat Riley, a far-gone consumptive Irishman, about forty-six years old, took China two globules of 8m of the preparation mentioned above May 10th, 1855.

On May 16th he was reported to have walked out in the street, after having been confined to his bed for twenty-six weeks. Before, however, he had lost speech entirely for twenty-four hours—from 4 P. M. to 4 P. M. the next day—and made signs that there was something catching in his throat. After that his speech returned unchanged. He never had any trouble about his speech before nor afterward, till it finally failed him altogether, when one and a half years later he died.

V.—Mr. C. Zimmer, the proprietor of the Chinine factory in Sachsenhausen, near Frankfort-on-the-Main, whose preparations of the alcaloids of Cinchona are celebrated for their purity and go all over the world, gave me the following facts observed in their manufacture, as the result of his long experience, which may be considered to be authoritative:

The workingmen are exposed to specific diseases, which represent themselves in two entirely different forms.

The first form is the so-called China-fever, to which the men are subjected who work in the mill grinding the bark, who, of course, must inhale the China-dust flying in the air. This fever consists of one very severe attack of chill and heat, with much nausea and vomiting, followed by prostration of strength for several days. This attack never returns in the same subject, who thereby gains an immunity from all future inhalations of China-dust. This China-fever, says Mr. Zimmer, is extremely rare, and he himself has seen only two cases in his experience, probably because the bark is now ground in a moist state. Mr. Chevalier, in a communication to the Academy of Science in Paris, says that this fever never occurs in the French factories, but is peculiar to Mr. Zimmer's manufactory.

The second form of disease, which especially affects persons of light complexion, who frequently are obliged for this reason to give up their occupation in the factory, is an eruption on the skin, which at first appears at the hands and in the face, and, if they persist in work, spreads all over the body, causing considerable pains, especially at night, and accompanied with swelling of the genitals. This eruption is very much like the bakers' itch. The susceptibility varies greatly. Persons with dark skin and hair are perfectly exempt, while those of fair complexion are easily affected, as mentioned above. This eruption shows itself in the various occupations of the men—in extraction, distillation, crystallization, and in the drying-rooms. As soon as the men relinquish their work in the factory and do something else they improve rapidly without medication, but the eruption breaks out again as soon as they resume work in the factory.

Mr. Zimmer adds that in the latter years no more diseases as described have occurred, because the men get more and more used to the effects of the bark and there is rarely a change made among them. Many men are not affected at all and some only slightly and temporarily.

## Figures and Tables

**Figure f1:**